# Comparison of the burden of anorexia nervosa in the Middle East and North Africa region between 1990 and 2019

**DOI:** 10.1186/s40337-022-00718-3

**Published:** 2022-12-10

**Authors:** Saeid Safiri, Maryam Noori, Seyed Aria Nejadghaderi, Seyed Ehsan Mousavi, Nahid Karamzad, Mark J. M. Sullman, Stephanie Pirotta, Gary S. Collins, Morteza Abdollahi, Ali-Asghar Kolahi

**Affiliations:** 1 Research Center for Integrative Medicine in Aging, Aging Research Institute, Tabriz University of Medical Sciences, Tabriz, Iran; 2 Department of Community Medicine, Faculty of Medicine, Tabriz University of Medical Sciences, Tabriz, Iran; 3 Student Research Committee, School of Medicine, Iran University of Medical Sciences, Tehran, Iran; 4 Systematic Review and Meta-Analysis Expert Group (SRMEG), Universal Scientific Education and Research Network (USERN), Tehran, Iran; 5 Student Research Committee, Tabriz University of Medical Sciences, Tabriz, Iran; 6 Department of Persian Medicine, School of Traditional Medicine, Tabriz University of Medical Sciences, Tabriz, Iran; 7 Nutrition Research Center, Department of Biochemistry and Diet Therapy, School of Nutrition and Food Sciences, Tabriz University of Medical Sciences, Tabriz, Iran; 8 Department of Life and Health Sciences, University of Nicosia, Nicosia, Cyprus; 9 Department of Social Sciences, University of Nicosia, Nicosia, Cyprus; 10 Health and Social Care Unit, School of Public Health and Preventive Medicine, Monash University, Melbourne, Australia; 11 Centre for Statistics in Medicine, Botnar Research Centre, NDORMS, University of Oxford, Oxford, UK; 12 NIHR Oxford Biomedical Research Centre, Oxford University Hospitals NHS Foundation Trust, Oxford, UK; 13 Social Determinants of Health Research Center, Shahid Beheshti University of Medical Sciences, Tehran, Iran

**Keywords:** Anorexia nervosa, Prevalence, Incidence, Epidemiology, Middle East and North Africa

## Abstract

**Background:**

Anorexia nervosa (AN) is a complex and heritable psychiatric disorder, which imposes significant mortality and morbidity on sufferers globally. We aimed to report the prevalence, incidence and disability-adjusted life-years (DALYs) attributable to AN in the Middle East and North Africa (MENA) region by age, sex and socio-demographic index (SDI), between 1990 and 2019.

**Methods:**

The disease burden attributable to AN was obtained for the 21 countries located in the MENA region between 1990 and 2019 using publicly available data from the Global Burden of Disease (GBD) 2019 study. All estimates were provided as counts and age-standardized rates per 100,000 population, along with 95% uncertainty intervals (UIs).

**Results:**

In 2019, the estimated age-standardised point prevalence and incidence rate of AN (per 100,000) in MENA were 49.3 (95% UI: 34.6–70.4) and 16.0 (11.3–22.0), which were 11.4% (7.3–15.4) and 5.9% (2.6–9.1) higher than in 1990, respectively. Furthermore, the regional age-standardised DALY rate was 10.6 (6.3–17.0) per 100,000 in 2019, which was 11.8% (5.2–19.1) higher than in 1990. In 2019, Kuwait [17.3 (10.3-27.9)] and Afghanistan [6.3 (3.7-10.3)] had the highest and lowest age-standardised DALY rates, respectively. In addition, Iran showed the largest increases in the age-standardised point prevalence [30.0% (24.1–36.2)], incidence [24.6% (18.6–30.4)] and DALY [30.5% (22.6–38.9)] rates between 1990 and 2019. In 2019, the number of prevalent cases and prevalence estimates peaked in the 15–19 age group for males and the 20–24 age group for females, with females having a higher number of cases and prevalence in all age groups. In 2019, the age-standardised DALY rates in MENA were higher than the global rates among males aged 10–34 years, but were lower than the global estimates among females in almost all age groups. In addition, the burden of AN was positively associated with the level of socio-economic development during the measurement period.

**Conclusions:**

The burden of AN in the MENA region increased between 1990 and 2019, which indicates that it is likely to become a more serious public health issue in the future. Up-to-date information about the epidemiological trends in the region would allow health policymakers to make informed and appropriate decisions to help address this issue.

**Plain English summary:**

The findings of the present study showed that the point prevalence and incidence rate of anorexia nervosa have increased in the Middle East and North Africa region between 1990 and 2019. The highest burden in 2019 was found in Kuwait, while Afghanistan had the lowest attributable burden. In addition, between 1990 and 2019 Iran had the largest increase in the point prevalence of anorexia nervosa. Also in 2019, anorexia nervosa was more prevalent in females and peaked in the 15–19 age group for males and the 20–24 age group for females. Furthermore, as the level of socioeconomic development increased, so did the burden attributable to anorexia nervosa.

**Supplementary Information:**

The online version contains supplementary material available at 10.1186/s40337-022-00718-3.

## Introduction

Anorexia nervosa (AN) is a complex and heritable mental health disorder, which is characterised by extreme weight loss following a severely restricted energy intake, intense fear of gaining weight or becoming fat and a disturbance in the way one’s body weight or shape is experienced [1]. Co-morbid psychiatric disorders, such as anxiety and depression, are commonly associated with AN [1]. AN can contribute to various complications like dizziness and fatigue in the acute phase and osteoporosis in the chronic phase [1]. Genetic, environmental, behavioural, psychological and developmental factors, such as female gender, personality traits, obsessive weight control and dysmaturity play roles in the pathogenesis of AN [1, 2]. Although psychotherapy and dietetic treatment are essential for AN recovery [3], there is currently a lack of effective treatments, which results in a very high relapse rate. As such, AN continues to have one of the highest morbidity rates among psychiatric disorders [4]. Understanding the burden of AN across the Middle East and North Africa (MENA) region will help inform the need for AN-specific health services and clinical training to better meet the needs of AN patients and caregivers, increase overall quality of life and help reduce the associated financial costs within this region. The prevalence rates and associated burden are increasing globally, including in the MENA region [5]. The global lifetime prevalence of AN in the general population has been reported to be 0.91%, with females being most vulnerable [5]. The global average age of AN onset is 17–22 years [6]. There was a global rise in AN, which resulted in the world-wide age-standardised disability-adjusted life-years (DALY) increasing from 8.5 in 1990 to 9.5 per 100,000 in 2017 [7]. Furthermore, the burden attributable to AN was found to be positively associated with a country’s level of development, irrespective of whether development was measured using the socio-demographic index (SDI) or the human development index [7].

Previous research reported the global burden of eating disorders using the Global Burden of Disease (GBD) 2017 [7]. In that study, Wu and colleagues reported the prevalence and DALYs attributable to eating disorders at the global, regional and national level, although incidence metrics were not provided [7]. Moreover, they reported the estimated annual percentage change between 1990 and 2017, while the present study focused on the percentage changes in the age-standardised rates of AN in the MENA region between 1990 and 2019 [7]. In addition, the methodologies used for estimating the burden of diseases in GBD 2019 have been substantially improved, since the previous iteration in 2017 [7]. In addition, the burden of mental disorders in the Eastern Mediterranean Region (EMR) have been reported for 1990–2013 and 1990–2015 using the 2013 and 2015 GBD data [8, 9], but no studies have focused specifically on the burden of AN in the MENA region. Moreover, due to the large sociocultural changes that are taking place in MENA, there are large number of potentially vulnerable adolescents and young adults [10, 11], and a large scale study of AN is needed in the MENA region. Therefore, this paper aimed to report the prevalence, incidence and DALYs attributable to AN in MENA by age, sex and SDI between 1990 and 2019.

## Methods

### Overview

In GBD 2019, the Institute for Health Metrics and Evaluation (IHME) collected data for 369 diseases and injuries, along with 87 risk factors from 1990 to 2019 [12, 13]. The MENA region includes: Afghanistan, Algeria, Bahrain, Egypt, Iran, Iraq, Jordan, Kuwait, Lebanon, Libya, Morocco, Oman, Palestine, Qatar, Saudi Arabia, Sudan, the Syrian Arab Republic, Tunisia, Turkey, the United Arab Emirates and Yemen. Additional information about the methodology used in the GBD studies to estimate the burden attributable to AN can be found in the GBD capstone article [12]. The data used in the current study can be obtained using the following links: https://vizhub.healthdata.org/gbd-compare/ and http://ghdx.healthdata.org/gbd-results-tool.

### Case definition and data sources

AN was defined using the four criteria outlined by the Diagnostic and Statistical Manual of Mental Disorders fourth edition, text revision (DSM-IV-TR): (a) Failure to maintain a body weight at or over the minimum normal weight for their age and height (e.g., weight loss which leads to a body weight < 85% of normal; or the failure to gain the expected weight over the growth period, reducing the body weight to < 85% of that expected); (b) An intense fear of gaining weight or becoming fat, even though underweight (DSM-5—generalisable to any behavior that interferes with weight gain); (c) Disturbed perception of their body weight or shape, undue influence of weight or shape on the individual’s self-evaluation, or the denial of the seriousness of their current low body weight; and (d) The occurrence of amenorrhea in postmenarchal females (i.e., the absence of at least three consecutive menstrual periods, which was removed in DSM-5) [12].

All studies which defined cases using the DSM or the International Classification of Diseases (ICD) diagnostic criteria were included in the GBD project. The codes used to identify the eligible cases for the DSM-IV-TR (307.1) and ICD-10 (F50.0–50.1) were used, but different editions of the DSM (DSM-III, DSM-III-R, DSM-IV, DSM-IV-TR, and DSM-5) and ICD (ICD-9 and ICD-10) were also accepted [12].

A systematic search of the literature was undertaken by IHME for GBD 2017 to identify studies reporting the prevalence, incidence, remission, and excess mortality associated with AN. Peer-reviewed databases (i.e., PsycInfo, Embase, and PubMed) and the grey literature were searched, and an expert was consulted. The GBD electronic database for mental disorders is updated biennially. A search of the grey literature and consultation with an expert were performed in GBD 2019 [12], with a new literature review planned for the following GBD iteration.

The inclusion criteria were: (1) Publication date from 1980 onwards; (2) AN cases ascertained using DSM or ICD diagnostic criteria; (3) the method contained enough information to evaluate the study quality and the characteristics of the participants were reported; and (4) Patients were obtained from the general population. Therefore, inpatients, pharmaceutical treatment subjects, case studies, veterans, and refugees were all excluded. There were no constraints regarding language. The mortality attributable to AN was obtained from the cause of death (COD) database, which contained centrally processed vital registration data. Further information on the data sources used by the GBD study can be found at the following link: https://ghdx.healthdata.org/gbd-2019/data-input-sources.

### Data processing and disease model

The following three methods were used for age and sex splitting, depending on the data format provided: (1) where possible, the estimates were divided by sex and age. In articles which reported AN prevalence for wide age groups by sex (e.g., 18–70 year old males and females, separately) or for less broad age groups without separating males and females (e.g., combined sexes for 15–30 year olds and 31–65 year olds) the sex ratio reported, along with the upper and lower limits of the uncertainty intervals (UI), were used to split the age-specific estimates into males and females; (2) The residual combined-sex estimates in the dataset were separated using a Meta-Regression with Bayesian priors, Regularization and Trimming (MR-BRT) analysis. MR-BRT network meta-analyses were used to calculate pooled sex ratios and uncertainty limits, with the values being used to produce separate sex estimates in each dataset. The male-to-female ratio was estimated to be 0.24 (95% UI: 0.05–0.43); and (3) Those reporting prevalence in age groups of 25 years or more were split into the required five-year age groups using the age pattern modelled by DisMod-MR 2.1. The DisMod-MR model contained no prior age split data [12]. Furthermore, several potential sources of bias between studies were examined (e.g., the use of ICD vs. DSM criteria and past year vs. point recall). However, none of the sources were found to significantly affect prevalence, and so bias adjustments were not used [12].

AN was not split according to severity level, but the lay description was feeling compelled to starve and/or exercising excessively in order to reduce body weight in a frail, feeble, and anxious individual. The disability weight for AN, sourced from the GBD disability weight survey, was 0.224 (0.150–0.312) [12].

DisMod-MR 2.1 was then used to model the epidemiological estimations for AN, using the GBD 2019 decomposition approach [12]. In each step of the decomposition approach, the new model was evaluated against the best model from GBD 2017, as well as the best model from the previous stage. Any significant differences were examined and explained, and where necessary alterations were made to the model priors or the dataset. If outliers were discovered, the methodology and quality of these studies were re-examined before deciding whether to retain the data or not [12]. In both the non-fatal and fatal models, it was assumed that there were no cases of AN before the age of 5 or after 50 years of age. The mortality estimates for AN were modelled using the Cause of Death Ensemble model (CODEm) and the eating disorders parent model. Fatalities were restricted to the ages of 5 and 49 years old, based on the advice of an expert and the prevalence patterns found in the non-fatal model. The covariates included in this model, as well as their direction, are reported in Additional file 4: Table S1. Further details on the modelling process have been previously reported [12].

There were two main changes in the GBD 2019 modelling strategy, compared to that used in GBD 2017:

1. In GBD 2017 the sex ratio was estimated using DisMod MR 2.1, as part of the prevalence modelling. In GBD 2019, MR-BRT was used by IHME to run a nested meta-regression analysis on the within study sex ratios to estimate a pooled sex ratio with 95% uncertainty intervals. Compared to GBD 2017, the male to female prevalence ratio increased slightly from 0.21 (0.14–0.40) to 0.24 (0.05–0.43).
2. In GBD 2019, globally there were eight new epidemiological data sources included from 12 different locations . Several of these studies were from locations where no data had previously existed.

### Compilation of results

The years of life lost (YLL) were estimated by multiplying the number of deaths in each age group by the remaining life expectancy in that age group (sourced from the GBD standard life table). The prevalence of AN was multiplied by its corresponding disability weight, extracted from the GBD Disability Weight assessment, to estimate the years lived with disability (YLD) [14]. The YLLs and YLDs were then summed to estimate the DALYs due to AN [12]. DALYs are defined as the years of healthy life lost, which is commonly used to measure the burden attributable to a particular health condition. In total, 1,000 draws were sampled at each computational step, including uncertainty from a number of different sources (e.g. input data, corrections of measurement error and estimates of residual non-sampling error), to generate 95% UIs for all estimates (the 25th and 975th values of the ordered draws).

Smoothing splines models were used to examine the relationship between the AN attributable DALYs and SDI for the 21 MENA countries [15]. The SDI, which ranges from 0 (least developed) to 1 (most developed), is based upon: 1) the 10 year average gross domestic output per capita; 2) mean level of education (years) in the population aged 15 and older; and 3) fertility rate among those under 25 years old [12]. R software (version 3.5.2) was used to conduct all statistical analyses.

## Results

### Burden of anorexia nervosa in the Middle East and North Africa region

In 2019, there were an estimated 327.9 thousand (95% UI: 229.9 to 467.2) prevalent cases of AN in the MENA region, with an age-standardised point prevalence of 49.3 (34.6–70.4) per 100,000 population, which was 11.4% (7.3 to 15.4) higher than in 1990 (Table 1 and Additional file 5: Table S2). AN accounted for 104.6 thousand (74.1–143.0) incident cases in 2019, with an age-standardised rate of 16.0 (11.3–22.0) per 100,000 population, which was 5.9% (2.6–9.1) higher than in 1990 (Table 1 and Additional file 6: Table S3). Also in 2019, the number of DALYs due to AN was 70.2 thousand (41.8–112.9), with an age-standardised rate of 10.6 (6.3–17.0) DALYs per 100,000 population, which had increased by 11.8% (5.2–19.1) since 1990 (Table 1 and Additional file 7: Table S4).

**Table 1:** Prevalent cases, incident cases and DALYs due to anorexia nervosa in 2019 and the percentage change in the age-standardised rates, 1990–2019

	Prevalence (95% UI)			Incidence (95% UI)			DALY (95% UI)		
	Counts	ASRs	PC in ASRs	Counts	ASRs	PC in ASRs	Counts	ASRs	PC in ASRs
	2019	2019	1990–2019	2019	2019	1990–2019	2019	2019	1990–2019
North Africa and Middle East	327,856	49.3	11.4	104,560	16	5.9	70,205	10.6	11.8
	(229,870, 467,180)	(34.6, 70.4)	(7.3, 15.4)	(74,084, 143,043)	(11.3, 22)	(2.6, 9.1)	(41,755, 112,883)	(6.3, 17)	(5.2, 19.1)
Afghanistan	13,100	29.9	− 10	5651	11.8	− 5.8	2787	6.3	− 8.6
	(9017, 18,744)	(20.7, 42.3)	(− 18.8, 0.4)	(3864, 8047)	(8.4, 16.6)	(− 14.9, 4.5)	(1579, 4614)	(3.7, 10.3)	(− 27.5, 16.2)
Algeria	21,309	49.5	4.6	6637	16.3	2.9	4552	10.6	4.6
	(14,898, 30,785)	(34.4, 71.6)	(− 6.4, 15.8)	(4692, 9127)	(11.3, 22.6)	(− 8.4, 14.7)	(2613, 7508)	(6.1, 17.4)	(− 15.1, 29.6)
Bahrain	901	64.5	6.4	224	19.6	3	192	13.8	6.4
	(632, 1267)	(44.5, 93.5)	(− 4.6, 18.4)	(159, 304)	(13.5, 27.3)	(− 9.6, 14.8)	(111, 298)	(7.8, 21.9)	(− 13.1, 28.9)
Egypt	50,811	47	19.4	17,544	15.8	12.2	10,876	10	20
	(35,230, 73,681)	(32.8, 67.9)	(6.4, 34.3)	(12,063, 24,461)	(10.9, 22)	(0.7, 24.7)	(6292, 17,521)	(5.8, 16.1)	(− 3.9, 50.3)
Iran (Islamic Republic of)	58,350	68.5	30	16,510	21.6	24.6	12,411	14.6	30.5
	(40,965, 82,330)	(47.9, 98.2)	(24.1, 36.2)	(11,781, 22,699)	(15.1, 30.1)	(18.6, 30.4)	(7298, 19,811)	(8.5, 23.8)	(22.6, 38.9)
Iraq	24,522	49	0.3	8338	16.2	0.4	5228	10.4	0.9
	(16,991, 35,906)	(33.9, 70.9)	(− 10.5, 10.7)	(5668, 11,786)	(11.2, 22.8)	(− 10, 11.5)	(2993, 8478)	(6, 16.9)	(− 18.4, 24.7)
Jordan	5785	43.2	5.9	2082	15.1	4.4	1245	9.3	6.2
	(4069, 8214)	(30.6, 60.7)	(− 4, 16.6)	(1451, 2952)	(10.6, 21.3)	(− 6.1, 15.5)	(723, 2035)	(5.4, 15.1)	(− 15.2, 31.7)
Kuwait	3869	78.7	12.1	820	21.5	4.3	849	17.3	11.1
	(2711, 5416)	(55.1, 112)	(1, 25.1)	(575, 1114)	(14.6, 30.5)	(− 7.1, 17.1)	(517, 1332)	(10.3, 27.9)	(− 6.2, 33.9)
Lebanon	2664	52.3	9	791	16.7	5.2	568	11.2	9.8
	(1862, 3808)	(36.6, 74.8)	(− 1.5, 20.3)	(552, 1117)	(11.5, 23.9)	(− 4.9, 17.1)	(335, 911)	(6.5, 18.1)	(− 10.8, 35.6)
Libya	3642	47.4	− 17.2	1143	15.9	− 11.7	777	10.1	− 17.1
	(2518, 5118)	(32.7, 66.9)	(− 25.3, − 7.1)	(802, 1582)	(11, 22.2)	(− 20.8, − 1)	(449, 1248)	(5.8, 16.4)	(− 32.1, 2.9)
Morocco	16,944	44.5	16.6	5689	15.2	9.9	3598	9.5	16.7
	(11,768, 23,675)	(30.9, 62.1)	(4.7, 28.9)	(3949, 7832)	(10.5, 20.9)	(− 2, 22.3)	(2042, 5857)	(5.4, 15.4)	(− 6.8, 45.7)
Oman	3218	57.9	5.7	842	18.6	4.9	687	12.4	5.6
	(2227, 4655)	(40, 84.5)	(− 4.6, 17)	(590, 1159)	(12.5, 26.1)	(− 7.2, 15.9)	(393, 1125)	(7.1, 20.1)	(− 12.3, 30.2)
Palestine	2203	38.4	15.2	847	13.9	10	469	8.2	15.3
	(1539, 3208)	(26.9, 54.7)	(3.9, 27.4)	(582, 1186)	(9.6, 19.5)	(− 1.5, 21.5)	(268, 765)	(4.7, 13.1)	(− 8.4, 44.2)
Qatar	2603	71.7	2.7	540	21.2	1.6	557	15.4	3.3
	(1766, 3741)	(50.2, 102.8)	(− 7.2, 13.9)	(384, 739)	(14.3, 30.5)	(− 8.9, 12.7)	(322, 894)	(8.9, 25)	(− 14.3, 22.1)
Saudi Arabia	26,753	61.1	2.6	6830	18.4	− 0.7	5887	13.4	4.3
	(18,794, 37,410)	(42.9, 86.3)	(− 8.1, 13.2)	(4833, 9523)	(12.7, 26)	(− 10.4, 9.6)	(3503, 9112)	(7.9, 20.7)	(− 13.2, 26.5)
Sudan	17,016	36	16.4	6700	13.3	9.9	3640	7.7	17
	(11,708, 24,718)	(25.2, 50.6)	(4.9, 30.8)	(4654, 9436)	(9.3, 18.5)	(− 1.2, 21.9)	(2090, 5924)	(4.5, 12.4)	(− 6.7, 46.8)
Syrian Arab Republic	6213	39.1	6.4	2435	14	3.3	1334	8.4	6.8
	(4298, 9002)	(27, 56.1)	(− 4.2, 17.5)	(1642, 3447)	(9.7, 19.6)	(− 7.9, 13.9)	(723, 2180)	(4.6, 13.6)	(− 13.7, 32.1)
Tunisia	5421	48.4	19.1	1671	16	10.9	1151	10.3	18.2
	(3825, 7632)	(34, 68.3)	(7.8, 33.6)	(1176, 2287)	(11.1, 22.2)	(0.3, 23.7)	(666, 1831)	(6, 16.6)	(− 4, 46.7)
Turkey	45,446	52.6	20.2	13,302	16.7	11.5	9768	11.3	20.5
	(31,970, 64,503)	(36.7, 74.4)	(7.7, 34.8)	(9388, 18,441)	(11.7, 23.4)	(− 0.9, 23.7)	(5795, 15,400)	(6.7, 18)	(− 0.3, 49.1)
United Arab Emirates	5640	66	− 15.4	1202	20.4	− 9.1	1199	14.2	− 15.2
	(3977, 7899)	(45.8, 95.6)	(− 24.9, − 5.7)	(873, 1631)	(13.9, 29.2)	(− 18.2, 2.9)	(705, 1902)	(8, 23.1)	(− 29.2, 2.2)
Yemen	11,112	31.2	− 6.6	4655	12.1	− 4	2358	6.6	− 6.1
	(7729, 15,785)	(21.8, 43.6)	(− 16.9, 3.4)	(3234, 6494)	(8.5, 16.9)	(− 13.7, 6)	(1349, 3770)	(3.8, 10.5)	(− 25.7, 19.6)

DALY = disability-adjusted-life-years; PC= percentage change. (Generated from data available from http://ghdx.healthdata.org/gbd-results-tool)

### Burden of anorexia nervosa at the national level

**Fig. 1:**
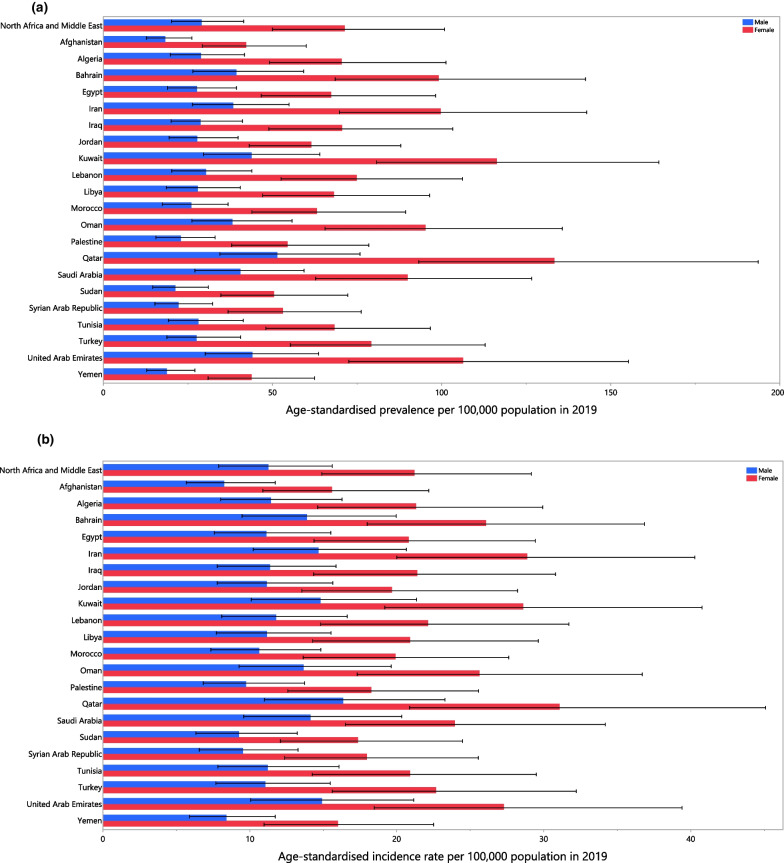

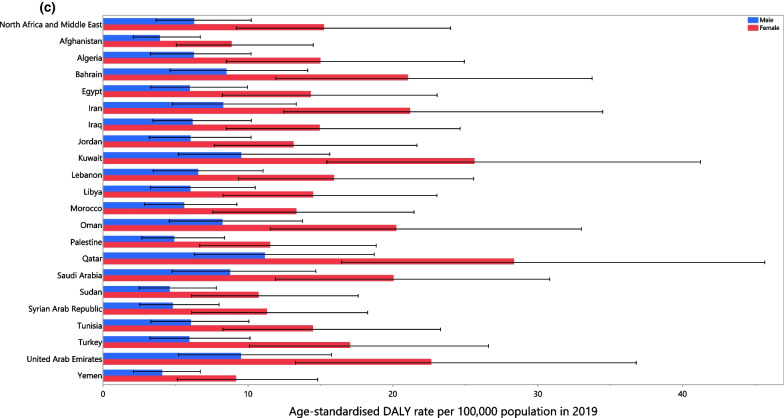
Age-standardised point prevalence (**a**), incidence (**b**), and DALYs (**c**) of anorexia nervosa (per 100,000 population) in the Middle East and North Africa region in 2019, by sex and country. DALY = disability-adjusted-life-years. (Generated from data available from http://ghdx.healthdata.org/gbd-results-tool)

The national age-standardised point prevalence of AN ranged in 2019 from 29.9 to 78.7 cases per 100,000 population across the MENA countries. Kuwait [78.7 (55.1–112.0), Qatar [71.7 (50.2–102.8)] and Iran [68.5 (47.9–98.2)] had the three highest age-standardised point prevalence rates in 2019. In contrast, Afghanistan [29.9 (20.7–42.3)], Yemen [31.2 (21.8–43.6)] and Sudan [36.0 (25.2 to 50.6)] had the lowest rates (Additional file 5: Table S2). Figure 1a presents the national age-standardised point prevalences of AN in 2019, by sex.

The national age-standardised incidence rate of AN varied from 11.8 to 21.6 cases per 100,000 population in 2019. The highest rates were observed in Iran [21.6 (15.1–30.1)], Kuwait [21.5 (14.6–30.5)] and Qatar [21.2 (14.3–30.5)], while the lowest rates were found in Afghanistan [11.8 (8.4–16.6)], Yemen [12.1 (8.5–16.9)] and Sudan [13.3 (9.3–18.5)] (Additional file 6: Table S3). The national age-standardised incidence rates of AN for 2019 are presented for males and females in Fig. 1b.

In 2019, the national age-standardised DALY rate of AN ranged from 6.3 to 17.3 cases per 100,000 population. The highest rates were observed in Kuwait [17.3 (10.3–27.9)], Qatar [15.4 (8.9–25.0)] and Iran [14.6 (8.5–23.8)]. Conversely, the lowest rates were seen in Afghanistan [6.3 (3.7–10.3)], Yemen [6.6 (3.8–10.5)] and Sudan [7.7 (4.5–12.4)] (Additional file 7: Table S4). The national age-standardised DALY rates in 2019 are presented for males and females in Fig. 1c.

Eight countries in the MENA region had increases in the age-standardised point prevalence of AN, between 1990 and 2019. Iran [30.0% (24.1–36.2)], Turkey [20.2% (7.7–34.8)] and Egypt [19.4% (6.4–34.3)] had the highest percentage changes in the age-standardised prevalence rate. In contrast, only Libya [−17.2% (−25.3 to −7.1)] and the United Arab Emirates [−15.4% (−24.9 to −5.7)] had decreased age-standardised point prevalence estimates (Additional file 1: Fig. S1 and Additional file 5: Table S2). Since 1990, the age-standardised incidence rate of AN has increased in Iran [24.6% (18.6 to 30.4)], Egypt [12.2% (0.7 to 24.7)] and Tunisia [10.9% (0.3 to 23.7)], while it has only decreased in Libya [−11.7% (−20.8 to −1.0)] (Additional file 2: Fig. S2 and Additional file 6: Table S3). In addition, Iran [30.5% (22.6 to 38.9)] had the largest increase in the age-standardised DALY rate between 1990 and 2019, whereas no county showed a decreased rate (Additional file 3: Fig. S3 and Additional file 7: Table S4).

### Age and sex patterns

**Fig. 2:**
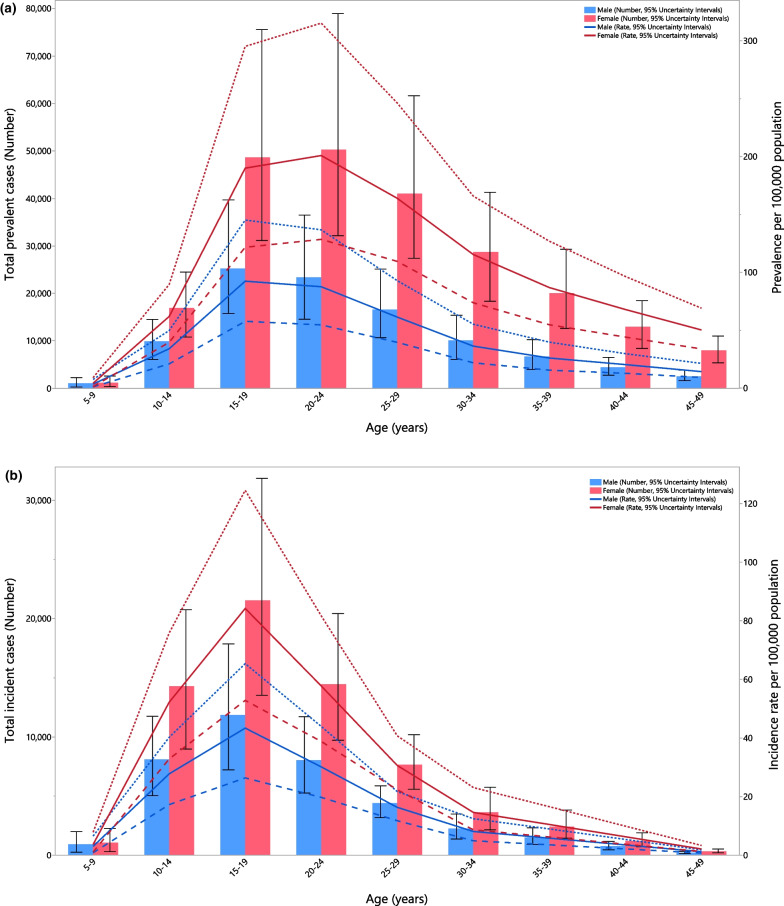

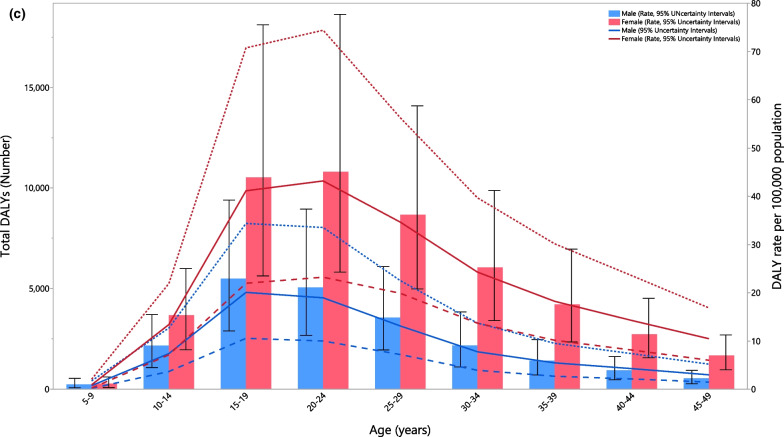
Numbers of prevalent cases and prevalence (**a**), number of incidence cases and incidence (**b**) and the number of DALYs and DALY rate (**c**) for anorexia nervosa per 100,000 population in the Middle East and North Africa region, by age and sex in 2019; Dotted and dashed lines indicate 95% upper and lower uncertainty intervals, respectively. DALY = disability-adjusted-life-years. (Generated from data available from http://ghdx.healthdata.org/gbd-results-tool)

In 2019, the number of prevalence cases and the point prevalence of AN reached its peak in the 15–19 age group for males and then decreased with advancing age. A similar pattern was observed for females, but the number of prevalent cases and the point prevalence reached its peak in the 20–24 age group (Fig. 2a). In 2019, the corresponding number of incident cases and the incidence rate increased steeply with age, peaking in the 15–19 age group and then sharply decreased up to the 25–29 age group, followed by further decreases with age (Fig. 2b). Furthermore, the number of DALYs and the DALY rate peaked in the 15–19 age group for males and the 20–24 age group for females, followed by decreases with age (Fig. 2c). The prevalence, incidence and DALYs, in terms of both numbers and rates, were higher in females of all ages.

**Fig. 3:**
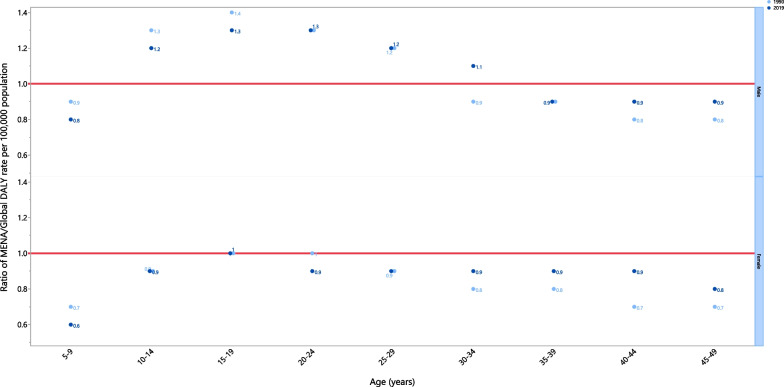
Ratio of the Middle East and North Africa region to the global anorexia nervosa DALY rate by age and sex, in 1990 and 2019. DALY = disability-adjusted-life-years. (Generated from data available from http://ghdx.healthdata.org/gbd-results-tool)

In 2019, the AN attributed DALY rate was lower than the global DALY rate (ratio of MENA/global DALY rate < 1) among males aged 5–9 and 35–49 years old, as well as females aged 5–14 and 20–49 years old. Females aged 15–19 years old had a DALY rate that was similar to the global rate (ratio of MENA/global DALY rate = 1), but there were no female age groups which had DALY rates that were higher than their corresponding global rate (ratio of MENA/global DALY rate > 1). Furthermore, males aged 10–34 years had higher DALY rates, with males aged 15–24 having a DALY rate that was 1.3 times higher than the corresponding global rate. Compared to 1990, in 2019 males had higher DALY rates in the 30–34 and 40–49 age groups and a lower DALY rate in the 5–19 age group. Also in comparison to 1990, the DALY rate in 2019 was lower for females aged 5–9 and 20–24 years old, and higher for those in the 30–49 age group (Fig. 3).

### Association with the socio-demographic Index (SDI)

**Fig. 4:**
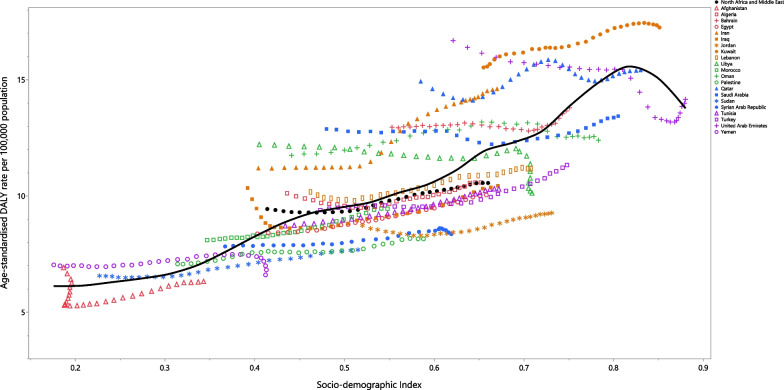
Age-standardised DALY rates of anorexia nervosa for 21 countries and territories, by SDI 1990–2019; Expected values based on the Socio-demographic Index and disease rates in all locations are shown as the black line. Each point shows the observed age-standardised DALY rate for each country during 1990–2019. DALY = disability-adjusted-life-years. SDI = Socio-demographic Index (Generated from data available from http://ghdx.healthdata.org/gbd-results-tool)

At the regional level, there was an almost perfect positive linear association between SDI and the DALY rate over the period 1990 to 2019. Kuwait and Iran had observed rates which were higher than the expected rates from 1990 to 2019, while Jordan and Tunisia had lower than expected rates for the entire measurement period. Afghanistan, Yemen, Sudan, Palestine, Syria, Morocco, Egypt, Iraq, Algeria, Lebanon, Libya, Oman, Saudi Arabia, Bahrain, Turkey and Qatar reached a lower than expected rate during the measurement period. After an initial decrease, and reaching a lower than expected rate, the United Arab Emirate reached a higher than expected rate during the measurement period (Fig. 4).

## Discussion

To the best of our knowledge, this is the first study using GBD 2019 data to report the most up-to-date information on the burden of disease attributable to AN in the MENA region between 1990 and 2019. In 2019, the age-standardised point prevalence of AN was 43.9, the incidence rate was 16.0, and the DALY rate was 10.6 (all per 100,000 population), which have increased by 11.4%, 5.9% and 11.8% since 1990, respectively. The AN prevalence rate peaked in the 15–19 age group for males and the 25–49 age group for females, with the rate being higher for females across all age-groups. In 2019, females in MENA had DALY rates that were lower than the global average in almost all age groups, while males aged 10–34 had DALY rates that were higher than the global average. Lastly, the burden of AN was positively associated with the developmental status of the MENA countries over the period 1990 to 2019.

According to the GBD 2019 project, in 2019 the age-standardised prevalence rate of eating disorders was 216.9 individuals per 100,000 population in MENA, which was higher than the global average of 174.0 cases per 100,000 [16]. In contrast, research using GBD 2017 data reported the prevalence of AN in the MENA region to be lower than the global average (32.8 vs. 43.8 per 100,000 population, respectively). That same study also reported that the annual increase in the DALY rate attributable to AN, over the past 30 years, was higher in MENA (0.69) than globally (0.45) [7].

A recent study conducted in Iran, as part of the Iranian Children and Adolescents' Psychiatric disorders (IRCAP) survey, examined feeding and eating disorders. The study enrolled 27,111 children and adolescents and used the DSM-5 criteria for disease definition [17]. In the multivariate analysis, the prevalence rate of AN was estimated to be 9.42 times higher in females than among males and 6.02 times higher in older adolescents than in early childhood, which is in accordance with our results. Another study conducted in Turkey used the EAT-40 test to screen 423 nursing students for eating disorders. Subjects who scored higher than 30 on the screening test were then assessed based on the DSV-IV eating disorders criteria [18]. The study estimated the prevalence rate of AN to be 0.5%. The most obvious reason for the higher rate found in the Turkish study is the fact that they only included female college students aged between 16 and 24 years, meaning that this result would not be generalisable to the general population.

The present study found that the burden of AN has increased in MENA, which may be explained by regional changes in specific risk factors. Although the precise pathogenesis of AN remains unclear, previous studies have pointed to several important biopsychosocial risk factors. Some genetic components [28, 29], particularly on chromosome 12 (rs4622308), clearly play a role in the pathogenesis of AN. There is also evidence of structural and functional alterations in the brain that seem to cause impaired mentalisation and defective reflective functioning in young patients affected by AN [30, 31]. However, the cultural and social trends related to beauty [32] could be by far the most prominent reason for the growing prevalence and incidence rates of AN in the MENA region. Furthermore, the differences in the AN estimates between MENA countries may be explained by cultural factors, such as internet addiction, distorted body image influenced by the media and unhealthy weight-loss diets [33].

Eating disorders were initially known as culture-bound syndromes. The notion that "slimness is beauty", which was mainly promoted by western countries [34, 35], led to a widespread adherence to unhealthy weight-control diets and body image distortions [36]. With time, the unhealthy emphasis on body shape and weight has extended to other cultures across the world [37]. Since 1990, countries in the MENA region have experienced rapid economic growth, urbanisation, and fundamental changes in family life, leading to western cultural transformations [38], which are a potential social risk factor for AN in this region. In addition, countries in the MENA region are experiencing decreases in neonatal deaths and rapid population growth [39]. The increasing number of adolescents and young adults could be contributing to the rising prevalence of AN in MENA.

AN causes life-threatening clinical complications and psychological disorders that may result in hospitalisation [19–22], and can even negatively affect the course of pregnancy and offspring growth. Previous studies have reported that maternal eating disorders are associated with low fetal growth, low birth weight, preterm labor and childhood wheezing [23–26]. Furthermore, females with eating disorders are at a higher risk of major depressive disorder during their lifetime [27]. Furthermore, male patients with AN usually delay seeking medical care and are often misdiagnosed by physicians, leading to more serious complications [40]. Therefore, it is also important to pay attention to male patients in order to avoid underestimating the burden caused by AN. Moreover, patients with AN are more prone to non-suicidal self-injuries, with a lifetime risk of 22% [41]. Affected patients are 1.7 times more likely to attempt suicide and 2.67 times more likely to die from suicide [42]. During the course of AN, the diagnosis can change to a different eating disorder, such as bulimia nervosa [43, 44]. These facts highlight the importance of having an early diagnosis, providing appropriate treatment, and having an adequate follow-up to help minimise the burden of AN.

The standard treatment for AN is nutritional rehabilitation, coupled with psychotherapy [45]. Unfortunately, relapse after restoring body weight and normal eating behavior is common [46], with relapse rates of up to 50% being reported following inpatient treatment [47]. Cognitive behavioral therapy (CBT) is a psychotherapy technique that helps patients restructure cognitive distortions about food and themselves and has been found to reduce the relapse rate [48, 49]. Furthermore, while the economic burden of AN has been estimated to be as high as 3,653–8,042 USD per person each year [50, 51], the use of evidence-based CBT treatments has been shown to lower the cost of AN at the population level. Research in Germany found that the use of CBT could reduce the AN-related direct healthcare costs associated with by about 1,005 million Euros and the indirect costs by around 289 million Euros each year [52]. Therefore, early diagnosis and the provision of timely and effective treatment is highly cost-effective. However, it should be noted that the psychological therapies provided in a western context may not be suitable in a different cultural setting [53], meaning that therapies may need to be adapted to accommodate different social norms and cultural beliefs [54, 55]. Furthermore, visiting mental health facilities is still a cultural taboo in the Middle East and many patients resort to self-diagnosis and self-medication [56, 57], often seeking help from herbal remedies or alternative medicine [58, 59]. This prevents patients from receiving timely and effective treatment, thereby increasing healthcare costs. Therefore, implementing effective interventions aimed at normalising mental health issues should be a top priority for health policy makers in the MENA region.

It is now evident that the actual burden of AN is grossly underestimated. Special attention needs to be given to high risk groups (e.g., children, adolescents, and pregnant women), such as raising awareness about eating disorders and breaking the cultural stigma associated with seeking help for mental health issues. In addition, public health policies aimed at improving the information primary health care providers have regarding AN would reduce the probability of misdiagnosis and promote an evidence-based approach to treating AN, thereby reducing the associated costs. Although the GBD 2019 provides valuable information about the burden of AN, studies that reported the burden of eating disorders in MENA are rare, making it difficult for policymakers to formulate appropriate evidence-based policies. Therefore, additional scientifically valid studies are needed in MENA to more accurately understand the attributable burden of AN in the MENA region.

This papers is the first to report the prevalence and burden of disease attributed to AN within the MENA countries. In recent years, several global, regional, and national studies have reported the burden of eating disorders and their associated patterns [16, 60–63]. However, to our knowledge no study has comprehensively and exclusively addressed the burden of AN in the MENA region. A number of epidemiological studies have reported the prevalence of AN in specific age and sex groups and in certain settings within specific MENA countries [17, 18, 64–71]. However, these studies mainly utilised eating attitude tests (EAT) to investigate eating behaviour and thus did not met the inclusion criteria of the GBD study [64, 65, 70–73]. The current research also has several limitations. The methodology used in GBD 2019 has several fundamental limitations, which have also been reported in previous GBD publications [12, 13]. Firstly, due to the scarcity of reliable and relevant studies in the MENA region, DisMod-MR 2.1 was used to map the epidemiological estimates for the areas with little or no data. For this purpose, DisMod-MR 2.1 produces estimates using the available data from surrounding countries [12]. Thus, these estimates should be interpreted with some caution, especially for low-income countries like Afghanistan, Sudan, and Yemen, where recent wars and social unrest have had devastating effects on their public health systems. Secondly, most of the studies conducted in MENA used EAT, which is a screening tool and cannot be used to establish a diagnosis. These studies were therefore not included in the GBD estimation process. Thirdly, older studies used previous versions of the DSM, which have a lower diagnostic yield and may have led to an underestimation of the number of cases. Fourthly, approximately 60% of deaths following AN occur as a consequence of clinical complications [22] (e.g., cardiovascular impairments, infectious diseases, and even organ failure), which do not result in AN being named as a cause of death, leading to the underestimation of the disease burden. Fifthly, AN has a broad spectrum of severity, ranging from mild clinical presentations to life-threatening events. In this case, the disability weight set for AN may not reflect the true spectrum of the disease severity and thus modifying the AN disability weights in future GBD iterations is highly recommended. Sixthly, the present study only reported the burden of AN in the MENA region, while the attributable burden of other eating disorders (e.g., bulimia nervosa or binge-eating disorder) will be reported in a separate article.

## Conclusions

The burden of AN in the MENA region was higher in 2019 than in 1990. Considering the population growth and rapid changes in the frequency of several leading risk factors, AN is likely to become a more serious public health issue in the near future. This highlights the urgent need to allocate more resources to researching AN and other eating disorders within this region, helping to inform AN-specific policy developments in the MENA countries. Doing so will help to enable the development and implementation of tailored, evidence-based healthcare services, clinical guidelines and training for local healthcare professionals to reduce the overall physical, emotional and financial costs associated with AN in the MENA countries.

## Supplementary Information

**Additional file 1**: **Fig. S1** The percentage change in the age-standardised prevalence of anorexia nervosa in the Middle East and North Africa region from 1990 to 2019, by sex and country. (Generated from data available from http://ghdx.healthdata.org/gbd-results-tool).

**Additional file 2**: **Fig. S2** The percentage change in the age-standardised incidence of anorexia nervosa in the Middle East and North Africa region from 1990 to 2019, by sex and country. (Generated from data available from http://ghdx.healthdata.org/gbd-results-tool).

**Additional file 3**: **Fig. S3** The percentage change in the age-standardised DALYs of anorexia nervosa in the Middle East and North Africa region from 1990 to 2019, by sex and country. DALY= disability-adjusted-life-years. (Generated from data available from http://ghdx.healthdata.org/gbd-results-tool).

**Additional file 4**: **Table S1** Covariates applied to CODEm model for anorexia nervosa in the Global Burden of Disease Study 2019.

**Additional file 5**: **Table S2** Prevalence of anorexia nervosa in 1990 and 2019 for both sexes and the percentage change in the age-standardised rates (ASRs) per 100,000 in the North Africa and the Middle East region (Generated from data available from http://ghdx.healthdata.org/gbd-results-tool).

**Additional file 6**: **Table S3** Incidence of anorexia nervosa in 1990 and 2019 for both sexes and the percentage change in the age-standardised rates (ASRs) per 100,000 in the North Africa and the Middle East region (Generated from data available from http://ghdx.healthdata.org/gbd-results-tool).

**Additional file 7**: **Table S4** DALYs due to anorexia nervosa in 1990 and 2019 for both sexes and the percentage change in the age-standardised rates (ASRs) per 100,000 in the North Africa and the Middle East region DALY= disability-adjusted-life-years. (Generated from data available from http://ghdx.healthdata.org/gbd-results-tool).

## Data Availability

The data used for these analyses are all publicly available at http://ghdx.healthdata.org/gbd-results-tool.
