# Supplementary figures and images for: Comparison of the burden of anorexia nervosa in the Middle East and North Africa region between 1990 and 2019
Source: J Eat Disord. 2022 Dec 10;10:192. doi: 10.1186/s40337-022-00718-3 (PMC9738022; doi:10.1186/s40337-022-00718-3)

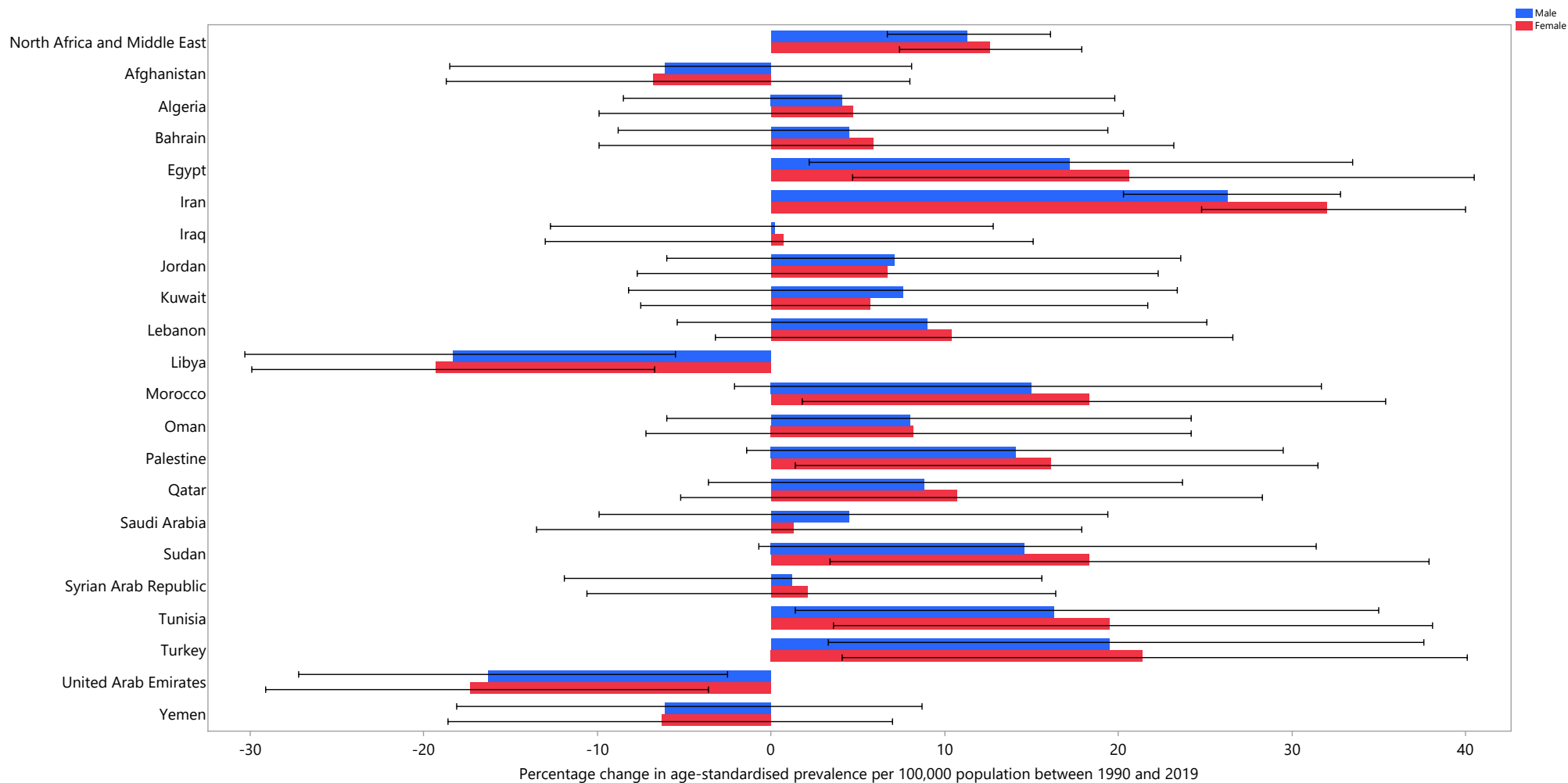

Supplement: Supplementary file 1 — Additional file 1: Fig. S1 The percentage change in the age-standardised prevalence of anorexia nervosa in the Middle East and North Africa region from 1990 to 2019, by sex and country. (Generated from data available from http://ghdx.healthdata.org/gbd-results-tool). [file 40337_2022_718_MOESM1_ESM.pdf]

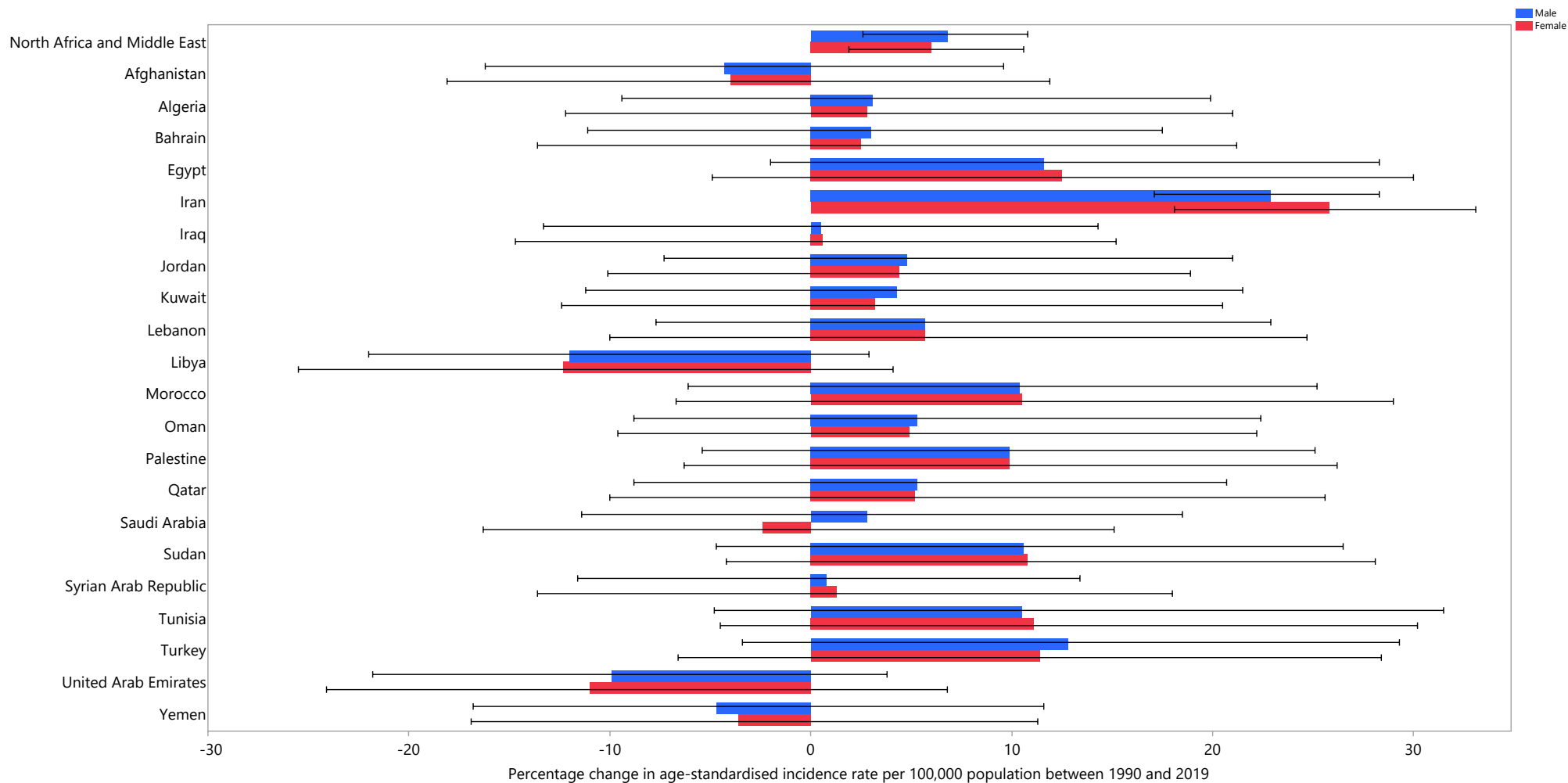

Supplement: Supplementary file 2 — Additional file 2: Fig. S2 The percentage change in the age-standardised incidence of anorexia nervosa in the Middle East and North Africa region from 1990 to 2019, by sex and country. (Generated from data available from http://ghdx.healthdata.org/gbd-results-tool). [file 40337_2022_718_MOESM2_ESM.pdf]

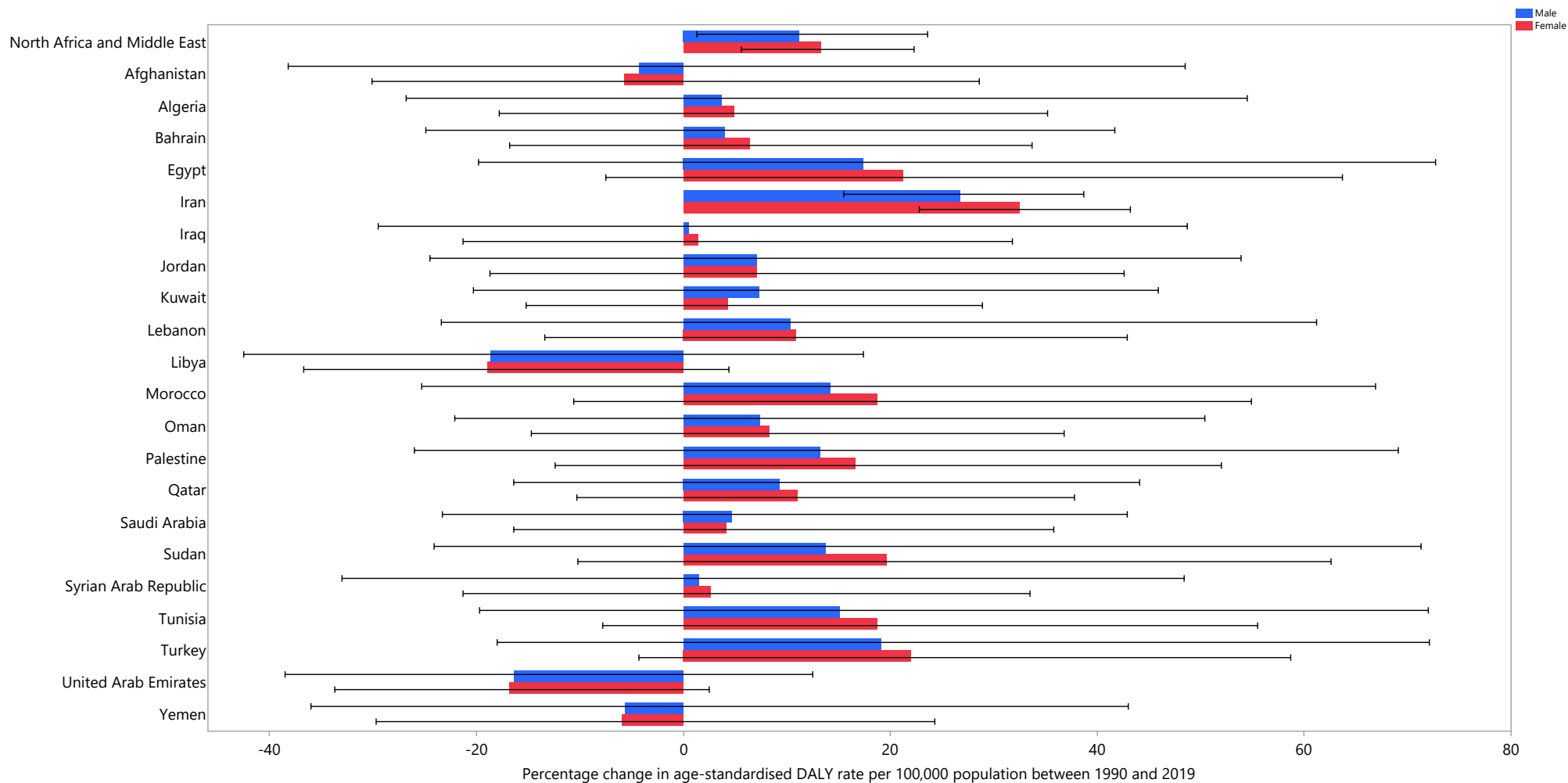

Supplement: Supplementary file 3 — Additional file 3: Fig. S3 The percentage change in the age-standardised DALYs of anorexia nervosa in the Middle East and North Africa region from 1990 to 2019, by sex and country. DALY= disability-adjusted-life-years. (Generated from data available from http://ghdx.healthdata.org/gbd-results-tool). [file 40337_2022_718_MOESM3_ESM.pdf]
