# Supplementary material for: Comparison of the burden of anorexia nervosa in the Middle East and North Africa region between 1990 and 2019
Source: J Eat Disord. 2022 Dec 10;10:192. doi: 10.1186/s40337-022-00718-3 (PMC9738022; doi:10.1186/s40337-022-00718-3)
Supplement: Supplementary file 4 — Additional file 4: Table S1 Covariates applied to CODEm model for anorexia nervosa in the Global Burden of Disease Study 2019. [file 40337_2022_718_MOESM4_ESM.docx]

| **Table S1: Selected covariates for the CODEm models in the anorexia nervosa estimation process** | | |
| --- | --- | --- |
| Level | Covariate | Direction |
| 1 | Education (years per capita) | + |
|  | Log LDI (I$ per capita) | + |
|  | Age- and sex-specific SEV for child underweight | - |
|  | Sanitation (proportion with access) | + |
|  | Maternal education (years per capita) | + |
| 2 | Healthcare access and quality index | - |
| 3 | Socio-demographic Index | + |
