# Supplementary material for: Comparison of the burden of anorexia nervosa in the Middle East and North Africa region between 1990 and 2019
Source: J Eat Disord. 2022 Dec 10;10:192. doi: 10.1186/s40337-022-00718-3 (PMC9738022; doi:10.1186/s40337-022-00718-3)
Supplement: Supplementary file 5 — Additional file 5: Table S2 Prevalence of anorexia nervosa in 1990 and 2019 for both sexes and the percentage change in the age-standardised rates (ASRs) per 100,000 in the North Africa and the Middle East region (Generated from data available from http://ghdx.healthdata.org/gbd-results-tool). [file 40337_2022_718_MOESM5_ESM.docx]

| **Table S2: Prevalence of anorexia nervosa in 1990 and 2019 and the percentage change in the age-standardised rates (ASRs) per 100,000 in the Middle East North and Africa region**  **(Generated from data available from http://ghdx.healthdata.org/gbd-results-tool)** | | | | | |
| --- | --- | --- | --- | --- | --- |
|  | **1990** | | **2019** | | **Percentage change in ASRs per 100,000** |
|  | **No (95% UI)** | **ASRs per 100,000 (95% UI)** | **No (95% UI)** | **ASRs per 100,000 (95% UI)** |  |
| **North Africa and Middle East** | **166805 (115382 , 243950)** | **44.3 (31 , 63.5)** | **327856 (229870 , 467180)** | **49.3 (34.6 , 70.4)** | **11.4 (7.3 , 15.4)** |
| **Afghanistan** | **3909 (2673 , 5771)** | **33.2 (23.2 , 47.9)** | **13100 (9017 , 18744)** | **29.9 (20.7 , 42.3)** | **-10 (-18.8 , 0.4)** |
| **Algeria** | **13383 (9093 , 19738)** | **47.3 (32.8 , 68.1)** | **21309 (14898 , 30785)** | **49.5 (34.4 , 71.6)** | **4.6 (-6.4 , 15.8)** |
| **Bahrain** | **367 (250 , 542)** | **60.6 (41.6 , 89.2)** | **901 (632 , 1267)** | **64.5 (44.5 , 93.5)** | **6.4 (-4.6 , 18.4)** |
| **Egypt** | **23783 (16271 , 35098)** | **39.3 (27.3 , 57.4)** | **50811 (35230 , 73681)** | **47 (32.8 , 67.9)** | **19.4 (6.4 , 34.3)** |
| **Iran (Islamic Republic of)** | **33274 (22984 , 48141)** | **52.7 (36.9 , 74.5)** | **58350 (40965 , 82330)** | **68.5 (47.9 , 98.2)** | **30 (24.1 , 36.2)** |
| **Iraq** | **9093 (6197 , 13320)** | **48.9 (33.8 , 70.3)** | **24522 (16991 , 35906)** | **49 (33.9 , 70.9)** | **0.3 (-10.5 , 10.7)** |
| **Jordan** | **1813 (1238 , 2700)** | **40.8 (28.1 , 58.9)** | **5785 (4069 , 8214)** | **43.2 (30.6 , 60.7)** | **5.9 (-4 , 16.6)** |
| **Kuwait** | **1493 (1032 , 2094)** | **70.2 (48.8 , 98.1)** | **3869 (2711 , 5416)** | **78.7 (55.1 , 112)** | **12.1 (1 , 25.1)** |
| **Lebanon** | **1596 (1104 , 2290)** | **47.9 (33.4 , 68.1)** | **2664 (1862 , 3808)** | **52.3 (36.6 , 74.8)** | **9 (-1.5 , 20.3)** |
| **Libya** | **2659 (1808 , 3913)** | **57.3 (39.9 , 82)** | **3642 (2518 , 5118)** | **47.4 (32.7 , 66.9)** | **-17.2 (-25.3 , -7.1)** |
| **Morocco** | **10897 (7529 , 16067)** | **38.2 (26.6 , 55.2)** | **16944 (11768 , 23675)** | **44.5 (30.9 , 62.1)** | **16.6 (4.7 , 28.9)** |
| **Oman** | **1139 (779 , 1644)** | **54.8 (37.5 , 79.1)** | **3218 (2227 , 4655)** | **57.9 (40 , 84.5)** | **5.7 (-4.6 , 17)** |
| **Palestine** | **736 (507 , 1081)** | **33.3 (23.3 , 47.2)** | **2203 (1539 , 3208)** | **38.4 (26.9 , 54.7)** | **15.2 (3.9 , 27.4)** |
| **Qatar** | **368 (254 , 523)** | **69.8 (48.3 , 99.9)** | **2603 (1766 , 3741)** | **71.7 (50.2 , 102.8)** | **2.7 (-7.2 , 13.9)** |
| **Saudi Arabia** | **10845 (7517 , 15939)** | **59.5 (41.1 , 86.7)** | **26753 (18794 , 37410)** | **61.1 (42.9 , 86.3)** | **2.6 (-8.1 , 13.2)** |
| **Sudan** | **6709 (4587 , 9705)** | **30.9 (21.4 , 44.1)** | **17016 (11708 , 24718)** | **36 (25.2 , 50.6)** | **16.4 (4.9 , 30.8)** |
| **Syrian Arab Republic** | **5143 (3506 , 7621)** | **36.7 (25.5 , 52.9)** | **6213 (4298 , 9002)** | **39.1 (27 , 56.1)** | **6.4 (-4.2 , 17.5)** |
| **Tunisia** | **3833 (2647 , 5540)** | **40.6 (28.4 , 58.6)** | **5421 (3825 , 7632)** | **48.4 (34 , 68.3)** | **19.1 (7.8 , 33.6)** |
| **Turkey** | **29456 (20226 , 43943)** | **43.8 (30.3 , 64.3)** | **45446 (31970 , 64503)** | **52.6 (36.7 , 74.4)** | **20.2 (7.7 , 34.8)** |
| **United Arab Emirates** | **1713 (1157 , 2479)** | **78 (53.2 , 113)** | **5640 (3977 , 7899)** | **66 (45.8 , 95.6)** | **-15.4 (-24.9 , -5.7)** |
| **Yemen** | **4483 (3074 , 6425)** | **33.4 (23.1 , 47.5)** | **11112 (7729 , 15785)** | **31.2 (21.8 , 43.6)** | **-6.6 (-16.9 , 3.4)** |
