# Supplementary material for: Comparison of the burden of anorexia nervosa in the Middle East and North Africa region between 1990 and 2019
Source: J Eat Disord. 2022 Dec 10;10:192. doi: 10.1186/s40337-022-00718-3 (PMC9738022; doi:10.1186/s40337-022-00718-3)
Supplement: Supplementary file 6 — Additional file 6: Table S3 Incidence of anorexia nervosa in 1990 and 2019 for both sexes and the percentage change in the age-standardised rates (ASRs) per 100,000 in the North Africa and the Middle East region (Generated from data available from http://ghdx.healthdata.org/gbd-results-tool). [file 40337_2022_718_MOESM6_ESM.docx]

| **Table S3: Incidence of anorexia nervosa in 1990 and 2019 and the percentage change in the age-standardised rates (ASRs) per 100,000 in the Middle East and North Africa region**  **(Generated from data available from http://ghdx.healthdata.org/gbd-results-tool)** | | | | | |
| --- | --- | --- | --- | --- | --- |
|  | **1990** | | **2019** | | **Percentage change in ASRs per 100,000** |
|  | **No (95% UI)** | **ASRs per 100,000 (95% UI)** | **No (95% UI)** | **ASRs per 100,000 (95% UI)** |  |
| **North Africa and Middle East** | **62535 (43189 , 86804)** | **15.2 (10.7 , 21)** | **104560 (74084 , 143043)** | **16 (11.3 , 22)** | **5.9 (2.6 , 9.1)** |
| **Afghanistan** | **1687 (1133 , 2399)** | **12.5 (8.8 , 17.5)** | **5651 (3864 , 8047)** | **11.8 (8.4 , 16.6)** | **-5.8 (-14.9 , 4.5)** |
| **Algeria** | **4992 (3343 , 7014)** | **15.8 (10.9 , 22)** | **6637 (4692 , 9127)** | **16.3 (11.3 , 22.6)** | **2.9 (-8.4 , 14.7)** |
| **Bahrain** | **106 (73 , 147)** | **19 (12.9 , 27)** | **224 (159 , 304)** | **19.6 (13.5 , 27.3)** | **3 (-9.6 , 14.8)** |
| **Egypt** | **9134 (6302 , 12859)** | **14.1 (9.8 , 19.7)** | **17544 (12063 , 24461)** | **15.8 (10.9 , 22)** | **12.2 (0.7 , 24.7)** |
| **Iran (Islamic Republic of)** | **12278 (8496 , 17170)** | **17.3 (12.2 , 24.1)** | **16510 (11781 , 22699)** | **21.6 (15.1 , 30.1)** | **24.6 (18.6 , 30.4)** |
| **Iraq** | **3408 (2316 , 4866)** | **16.2 (11.3 , 22.7)** | **8338 (5668 , 11786)** | **16.2 (11.2 , 22.8)** | **0.4 (-10 , 11.5)** |
| **Jordan** | **721 (494 , 1015)** | **14.5 (10.2 , 20.2)** | **2082 (1451 , 2952)** | **15.1 (10.6 , 21.3)** | **4.4 (-6.1 , 15.5)** |
| **Kuwait** | **406 (281 , 561)** | **20.6 (14 , 28.9)** | **820 (575 , 1114)** | **21.5 (14.6 , 30.5)** | **4.3 (-7.1 , 17.1)** |
| **Lebanon** | **567 (387 , 811)** | **15.9 (11.1 , 22.6)** | **791 (552 , 1117)** | **16.7 (11.5 , 23.9)** | **5.2 (-4.9 , 17.1)** |
| **Libya** | **952 (638 , 1351)** | **18 (12.5 , 25.3)** | **1143 (802 , 1582)** | **15.9 (11 , 22.2)** | **-11.7 (-20.8 , -1)** |
| **Morocco** | **4232 (2930 , 6032)** | **13.8 (9.7 , 19.6)** | **5689 (3949 , 7832)** | **15.2 (10.5 , 20.9)** | **9.9 (-2 , 22.3)** |
| **Oman** | **385 (266 , 541)** | **17.8 (12.2 , 25.1)** | **842 (590 , 1159)** | **18.6 (12.5 , 26.1)** | **4.9 (-7.2 , 15.9)** |
| **Palestine** | **312 (212 , 440)** | **12.6 (8.8 , 17.5)** | **847 (582 , 1186)** | **13.9 (9.6 , 19.5)** | **10 (-1.5 , 21.5)** |
| **Qatar** | **93 (65 , 128)** | **20.9 (14.1 , 29.5)** | **540 (384 , 739)** | **21.2 (14.3 , 30.5)** | **1.6 (-8.9 , 12.7)** |
| **Saudi Arabia** | **3621 (2456 , 5036)** | **18.6 (12.8 , 25.7)** | **6830 (4833 , 9523)** | **18.4 (12.7 , 26)** | **-0.7 (-10.4 , 9.6)** |
| **Sudan** | **2863 (1988 , 4079)** | **12.1 (8.6 , 16.8)** | **6700 (4654 , 9436)** | **13.3 (9.3 , 18.5)** | **9.9 (-1.2 , 21.9)** |
| **Syrian Arab Republic** | **2141 (1449 , 3020)** | **13.5 (9.4 , 18.8)** | **2435 (1642 , 3447)** | **14 (9.7 , 19.6)** | **3.3 (-7.9 , 13.9)** |
| **Tunisia** | **1458 (994 , 2047)** | **14.4 (10 , 20.1)** | **1671 (1176 , 2287)** | **16 (11.1 , 22.2)** | **10.9 (0.3 , 23.7)** |
| **Turkey** | **10774 (7340 , 15311)** | **15 (10.5 , 21.1)** | **13302 (9388 , 18441)** | **16.7 (11.7 , 23.4)** | **11.5 (-0.9 , 23.7)** |
| **United Arab Emirates** | **421 (291 , 598)** | **22.4 (15.1 , 32.3)** | **1202 (873 , 1631)** | **20.4 (13.9 , 29.2)** | **-9.1 (-18.2 , 2.9)** |
| **Yemen** | **1940 (1327 , 2714)** | **12.6 (8.9 , 17.5)** | **4655 (3234 , 6494)** | **12.1 (8.5 , 16.9)** | **-4 (-13.7 , 6)** |
