# Supplementary material for: Comparison of the burden of anorexia nervosa in the Middle East and North Africa region between 1990 and 2019
Source: J Eat Disord. 2022 Dec 10;10:192. doi: 10.1186/s40337-022-00718-3 (PMC9738022; doi:10.1186/s40337-022-00718-3)
Supplement: Supplementary file 7 — Additional file 7: Table S4 DALYs due to anorexia nervosa in 1990 and 2019 for both sexes and the percentage change in the age-standardised rates (ASRs) per 100,000 in the North Africa and the Middle East region DALY= disability-adjusted-life-years. (Generated from data available from http://ghdx.healthdata.org/gbd-results-tool). [file 40337_2022_718_MOESM7_ESM.docx]

| **Table S4: DALYs due to anorexia nervosa in 1990 and 2019 and the percentage change in the age-standardised rates (ASRs) per 100,000 in the Middle East and North Africa region**  **(Generated from data available from http://ghdx.healthdata.org/gbd-results-tool)** | | | | | |
| --- | --- | --- | --- | --- | --- |
|  | **1990** | | **2019** | | **Percentage change in ASRs per 100,000** |
|  | **No (95% UI)** | **ASRs per 100,000 (95% UI)** | **No (95% UI)** | **ASRs per 100,000 (95% UI)** |  |
| **North Africa and Middle East** | **35678 (20676 , 57748)** | **9.4 (5.6 , 15)** | **70205 (41755 , 112883)** | **10.6 (6.3 , 17)** | **11.8 (5.2 , 19.1)** |
| **Afghanistan** | **818 (457 , 1386)** | **6.9 (4 , 11.3)** | **2787 (1579 , 4614)** | **6.3 (3.7 , 10.3)** | **-8.6 (-27.5 , 16.2)** |
| **Algeria** | **2869 (1625 , 4722)** | **10.1 (5.9 , 16.6)** | **4552 (2613 , 7508)** | **10.6 (6.1 , 17.4)** | **4.6 (-15.1 , 29.6)** |
| **Bahrain** | **78 (45 , 126)** | **13 (7.4 , 21.1)** | **192 (111 , 298)** | **13.8 (7.8 , 21.9)** | **6.4 (-13.1 , 28.9)** |
| **Egypt** | **5076 (2856 , 8163)** | **8.4 (4.8 , 13.3)** | **10876 (6292 , 17521)** | **10 (5.8 , 16.1)** | **20 (-3.9 , 50.3)** |
| **Iran (Islamic Republic of)** | **7104 (4120 , 11364)** | **11.2 (6.6 , 17.8)** | **12411 (7298 , 19811)** | **14.6 (8.5 , 23.8)** | **30.5 (22.6 , 38.9)** |
| **Iraq** | **1933 (1091 , 3201)** | **10.4 (5.9 , 16.9)** | **5228 (2993 , 8478)** | **10.4 (6 , 16.9)** | **0.9 (-18.4 , 24.7)** |
| **Jordan** | **390 (218 , 655)** | **8.7 (5 , 14.2)** | **1245 (723 , 2035)** | **9.3 (5.4 , 15.1)** | **6.2 (-15.2 , 31.7)** |
| **Kuwait** | **330 (196 , 537)** | **15.5 (9.2 , 25.1)** | **849 (517 , 1332)** | **17.3 (10.3 , 27.9)** | **11.1 (-6.2 , 33.9)** |
| **Lebanon** | **340 (193 , 565)** | **10.2 (5.8 , 16.7)** | **568 (335 , 911)** | **11.2 (6.5 , 18.1)** | **9.8 (-10.8 , 35.6)** |
| **Libya** | **569 (325 , 951)** | **12.2 (7.2 , 20.1)** | **777 (449 , 1248)** | **10.1 (5.8 , 16.4)** | **-17.1 (-32.1 , 2.9)** |
| **Morocco** | **2318 (1334 , 3781)** | **8.1 (4.7 , 13)** | **3598 (2042 , 5857)** | **9.5 (5.4 , 15.4)** | **16.7 (-6.8 , 45.7)** |
| **Oman** | **244 (138 , 403)** | **11.7 (6.6 , 19.3)** | **687 (393 , 1125)** | **12.4 (7.1 , 20.1)** | **5.6 (-12.3 , 30.2)** |
| **Palestine** | **157 (90 , 260)** | **7.1 (4.1 , 11.5)** | **469 (268 , 765)** | **8.2 (4.7 , 13.1)** | **15.3 (-8.4 , 44.2)** |
| **Qatar** | **79 (45 , 126)** | **14.9 (8.4 , 24)** | **557 (322 , 894)** | **15.4 (8.9 , 25)** | **3.3 (-14.3 , 22.1)** |
| **Saudi Arabia** | **2350 (1333 , 3857)** | **12.9 (7.4 , 20.9)** | **5887 (3503 , 9112)** | **13.4 (7.9 , 20.7)** | **4.3 (-13.2 , 26.5)** |
| **Sudan** | **1430 (811 , 2419)** | **6.6 (3.7 , 10.9)** | **3640 (2090 , 5924)** | **7.7 (4.5 , 12.4)** | **17 (-6.7 , 46.8)** |
| **Syrian Arab Republic** | **1099 (609 , 1799)** | **7.8 (4.4 , 12.6)** | **1334 (723 , 2180)** | **8.4 (4.6 , 13.6)** | **6.8 (-13.7 , 32.1)** |
| **Tunisia** | **824 (472 , 1385)** | **8.7 (5.1 , 14.4)** | **1151 (666 , 1831)** | **10.3 (6 , 16.6)** | **18.2 (-4 , 46.7)** |
| **Turkey** | **6333 (3576 , 10224)** | **9.4 (5.4 , 15.1)** | **9768 (5795 , 15400)** | **11.3 (6.7 , 18)** | **20.5 (-0.3 , 49.1)** |
| **United Arab Emirates** | **365 (209 , 586)** | **16.7 (9.4 , 26.7)** | **1199 (705 , 1902)** | **14.2 (8 , 23.1)** | **-15.2 (-29.2 , 2.2)** |
| **Yemen** | **948 (535 , 1618)** | **7 (4 , 11.6)** | **2358 (1349 , 3770)** | **6.6 (3.8 , 10.5)** | **-6.1 (-25.7 , 19.6)** |
